# Effects of Sodium-Glucose Cotransporter Inhibitor/Glucagon-Like Peptide-1 Receptor Agonist Add-On to Insulin Therapy on Glucose Homeostasis and Body Weight in Patients With Type 1 Diabetes: A Network Meta-Analysis

**DOI:** 10.3389/fendo.2020.00553

**Published:** 2020-08-19

**Authors:** Yoon Ji Kim, Seun Deuk Hwang, Soo Lim

**Affiliations:** ^1^Division of Endocrinology and Metabolism, Department of Internal Medicine, Mediplex Sejong Hospital, Incheon, South Korea; ^2^Division of Nephrology and Hypertension, Department of Internal Medicine, Inha University College of Medicine, Incheon, South Korea; ^3^Department of Internal Medicine, Seoul National University College of Medicine, Seoul National University Bundang Hospital, Seongnam, South Korea

**Keywords:** SGLT inhibitor, GLP-1 receptor agonist, type 1 diabetes, add on to insulin therapy, body weight, glycemic level

## Abstract

Many patients with type 1 diabetes (T1D) do not achieve the glycemic target goal with insulin treatment. In this study, we aimed to evaluate the efficacy and safety of add-on to insulin therapy in patients with T1D. We conducted direct and indirect network meta-analyses using Bayesian models and ranked hypoglycemic agents via mixed treatment comparison, using data from the CENTRAL, MEDLINE, EMBASE, and Science Citation Index Expanded databases. Randomized controlled trials (RCTs) involving patients with T1D treated with insulin and add-on metformin or sodium-glucose cotransporter inhibitors or glucagon-like peptide-1 receptor agonists from January 1970 to September 2019 were included in this study. Twenty-three RCTs with 5,151 subjects were divided into the following groups: insulin alone, insulin+metformin, insulin+canagliflozin, insulin+dapagliflozin, insulin+empagliflozin, insulin+sotagliflozin, insulin+liraglutide, and insulin+exenatide. HbA1c level in the insulin+sotagliflozin group was significantly lower than that in the insulin alone group (mean difference: −0.43, 95% credible interval: −0.62 to −0.23). Total daily insulin dose in the insulin+sotagliflozin group was significantly lower than that in the insulin alone group. Compared with that in the insulin alone group, body weight in the groups treated with insulin+add-on canagliflozin, sotagliflozin, and exenatide was significantly decreased by 4.5, 2.8, and 5.1 kg, respectively. Hypoglycemic episodes did not differ among the groups. In patients with T1D, insulin+sotagliflozin decreased the HbA1c level, daily insulin dose, and body weight without hypoglycemia compared with insulin monotherapy. Insulin+canagliflozin or insulin+exenatide was effective in reducing body weight compared with insulin alone. In conclusion, sotagliflozin treatment decreased not only the HbA1c levels and insulin dose but also the body weight without causing hypoglycemia in patients with T1D. Treatment with canagliflozin and exenatide effectively reduced body weight in patients with T1D. However, ketoacidosis associated with the use of SGLT inhibitors should be considered in these patients. Thus, our results suggest that sotagliflozin has a high probability of being ranked first as an adjunctive therapy to insulin in patients with T1D.

## Introduction

The incidence of type 1 diabetes (T1D) is continuously increasing. According to a report from the International Diabetes Federation (IDF), T1D currently affects 29 million adults worldwide (1). The IDF reported that the number of children and adolescents with T1D in 2017 was 1,106,500. Moreover, 132,600 patients are newly diagnosed with T1D every year. According to the T1D Exchange Registry data, in more than 70% of patients with T1D, glycated hemoglobin (HbA1c) levels lower than 7% was not achieved (2).

Compared with the treatment of type 2 diabetes (T2D) using various novel medications, that of T1D mostly depends on insulin. Several drugs have been investigated as an adjunct therapy for T1D, but the US FDA approved only pramlintide, which mimics a β-cell hormone that is co-secreted with insulin in the postprandial period, in 2005 (3). However, the effects of pramlintide on HbA1c level and weight changes are mild and unsatisfactory (4).

Metformin is the most studied oral antidiabetic drug used as an adjunct for T1D treatment. It suppresses hepatic gluconeogenesis and increases glucose uptake by muscles via the amplification of glucose transporter 4 (5). Treatment of T1D with metformin reportedly reduces insulin requirement and decreases body mass index (BMI), although the HbA1c level was similar to that of the placebo treatment (6, 7). Dipeptidyl peptidase-4 (DPP-4) inhibitors prolong the half-life of endogenous glucagon-like peptide-1 (GLP-1), which stimulates glucose-dependent insulin secretion and inhibits glucagon release. Although the effect of DPP-4 inhibitors in chronic T1D has not been elucidated, a study reported more than 20 IU reduction in daily insulin dose in newly diagnosed patients with T1D (4). GLP-1 receptor agonists (GLP-1RA), such as liraglutide and exenatide, are also reported to reduce insulin dose and decrease body weight in patients with T1D. However, their effect on HbA1c was not consistent among studies (8–15). Sodium-glucose cotransporter-2 (SGLT-2) inhibitors, which reduce glucose reabsorption in the proximal tubules of the kidney, are one of the most attractive drugs for T2D owing to their beneficial effects on cardiovascular and renal functions (16, 17). Compared with that of insulin administration alone, administration of SGLT-2 inhibitors, such as dapagliflozin, empagliflozin, and canagliflozin, decreased HbA1c level, body weight, and insulin requirement in patients with T1D (18–23). Recently, researchers have focused on SGLT-1/2 co-inhibitors because they can simultaneously inhibit the absorption of sugars in the kidneys and intestine (24). Garg et al. showed that sotagliflozin, an SGLT-1/2 co-inhibitor, decreased HbA1c level in patients with T1D (1).

Several trials with GLP-1 RA, SGLT-2 inhibitors, or SGLT-1/2 co-inhibitors as adjuncts to insulin therapy for T1D have been conducted. However, no conclusive suggestion has been made because of the following reasons: the glucose-lowering efficacy of these agents was not satisfactory; the trials had a small sample size; and most importantly, these agents were only compared with insulin and not with other agents. Therefore, the aim of our study was to identify the most efficient drug as an add-on to insulin therapy in patients with T1D through a network meta-analysis.

## Materials and Methods

### Ethics Statement

The results are presented in accordance with the guidelines of Preferred Reporting Items for Systematic Reviews and Network Meta-Analyses statement (NMA Checklist) (25). All analyses were conducted using previously published studies; therefore, ethical approval and patient consent were not required.

### Data Sources, Searches, and Inclusion and Exclusion Criteria

We performed a comprehensive search of the following databases, from the time of the inception of each database until September 2019: MEDLINE (via PubMed), EMBASE, CINAHL, Web of Science, and the Cochrane Central Register of Controlled Trials in the Cochrane Library. The following terms were used to identify RCTs: Metformin or Sodium-Glucose Transport Proteins or Sodium-Glucose Transporter 2 or Sodium-Glucose Transporter 2 Inhibitors or SGLT1/2 inhibitor or Exenatide or Liraglutide and Diabetes Mellitus, Type 1. Detailed search terms are provided in Supplementary Information 1.

**Figure 1:**
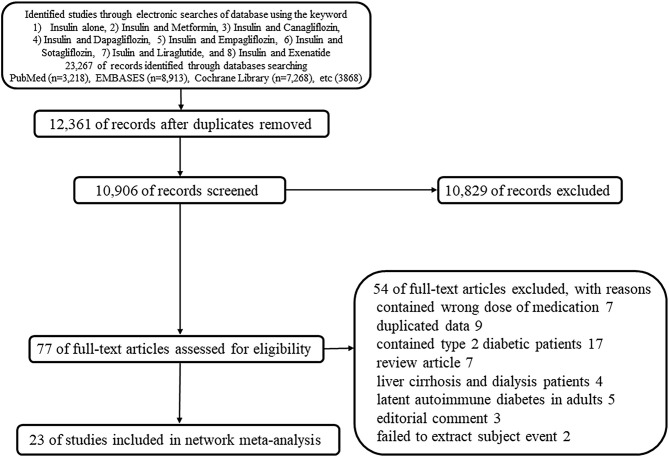
Flow diagram of the current systematic review (PRISMA Flow Diagram).

The following studies were included in the review: RCTs, reviews, observational studies, and clinical trials. The search was limited to human studies but was not restricted to any particular language or publication date. Reference lists from all available review articles and RCTs were searched manually (Figure 1).

### Study Selection

**Figure 2:**
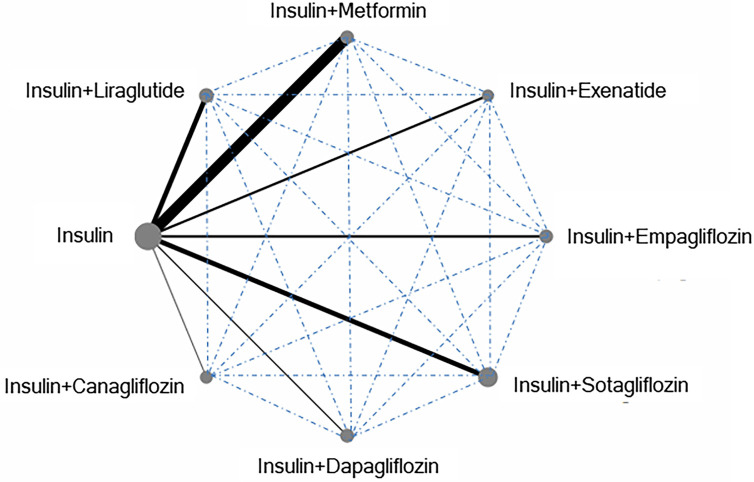
Network flow among each intervention based on HbA1c data.

The abstracts and full texts obtained were independently checked by two researchers (YJK and SDH). Any disagreements were resolved through discussions and consultations with another researcher. The inclusion criteria for studies used in the analysis were as follows: (1) randomized controlled studies; (2) studies referring to at least two of the following eligible antidiabetic medications: placebo, metformin, canagliflozin, dapagliflozin, empagliflozin, sotagliflozin, liraglutide, and exenatide; and (3) studies that reported one or more of the primary or secondary outcomes (Figure 2). Treatments with direct comparisons are linked with a line, whose thickness corresponds to the number of trials evaluating that comparison. For example, when insulin is used as a reference, the line comparing insulin and metformin is the boldest indicating that these two interventions are most evaluated, while being solid line (rather than dotted) indicates a direct evaluation. Conversely, dotted lines indicate indirect connections expressed using direct comparison and indirect comparison due to lack of head-to-head study. For example, line comparing insulin sotagliflozin and insulin dapagliflozin indicates no direct study; the indirect connection in the network was, therefore, was calculated. Trials that recruited patients with T2D or latent autoimmune diabetes were excluded.

### Risk of Bias Assessment

Two researchers (YJK and SDH) independently assessed the risk of bias of each trial using the Cochrane Collaboration's Risk of Bias tool (26). The risk of bias was assessed during the generation of random sequence, concealment of allocation, blinding of participants and personnel, blinding of outcome assessment, analysis of incomplete outcome data, selective reporting, and in other areas. All these judgments were categorized as “yes” (low risk of bias) or “unclear” or “no” (high risk of bias) (Supplementary Figures 1, 2) (26, 27).

### Quality of Evidence Assessment

We assessed the overall evidence quality for the primary outcomes using an adapted Grading of Recommendations Assessment, Development, and Evaluation approach (28). The evidence quality for a specific outcome was based on performance vs. limitations of the study design, inconsistency of results, indirectness of evidence, imprecision of results, and publication bias of all studies measuring a particular outcome. The overall evidence quality for the outcome was determined by combining assessments from all domains (Supplementary Figure 3) (29).

### Outcome Measures

We aimed to determine the efficacy of eligible medications on changes in HbA1c level (mean ± standard deviation [from the baseline to endpoint]) as the primary outcome. The occurrence of hypoglycemia, reduction in insulin daily dose, and change in body weight were determined as secondary outcomes. Moreover, the potential for adverse outcomes associated with these medications, including diabetic ketoacidosis, heart failure, stroke, diarrhea, pancreatitis, renal event, urinary tract infection, and genital infection, were investigated.

### Statistical Analysis

Bayesian network meta-analysis was performed to compare the efficacy of eight types of diabetes treatments in terms of HbA1c and weight reduction outcomes and adverse outcomes in patients with type 1 diabetes. Direct and indirect network meta-analyses were performed using Bayesian models, and the different agents were ranked by mixed treatment comparison (GeMTC) and using Stata version 13 (StataCorp) (30–32). The relative ranking probability of each treatment was estimated, and the treatment hierarchy of competing interventions was obtained using rankograms, surface under the cumulative ranking curves, and mean ranks. The network meta-analysis was performed on studies evaluating multiple treatments, which allows the estimation of pooled effects within each treatment (33). For multi-arm trials, correlations among the treatment effects among arms were included in the investigations. Studies with *j*+1 treatment arms were based on comparison of the treatment effects with the reference treatment effects through multivariate normal distribution, whereas treatment-as-usual studies were based on the homogeneity among study variances across treatments (34, 35). Inconsistency tests, homogeneity analysis, and sensitivity analysis were performed using the node analysis method in R software (The R Foundation for Statistical Computing c/o Institute for Statistics and Mathematics, Vienna, Austria). The results of inconsistency tests were assessed according to the Bayesian *p-*value, where the results with *p* < 0.5 were considered an evidence for the existence of significant inconsistency (36, 37). An *I*^2^ test was performed (*I*^2^ > 50% indicated significant heterogeneity) to assess homogeneity. Furthermore, a sensitivity analysis was conducted by comparing the differences between fixed-effect and random-effect models. The clinical outcome indicators were evaluated using the mean difference or odds ratio (OR) with a 95% credible interval (CrI) (mean difference for continuous outcomes and OR for binary outcomes) (34, 38). When a loop connected three treatments, it was possible to evaluate the inconsistency between direct and indirect evidence (39). We also used the node-splitting method to calculate the inconsistency of the model, which separated evidence for a particular comparison into direct and indirect evidence (37). Subsequently, the agreement between direct and indirect evidence was evaluated, and its Bayesian *p*-value was obtained. Sensitivity analyses were carried out using the same methods, after the omission of data obtained from specific studies (studies with a small number of patients and events in a specific treatment arm and studies with a large population that may dominate the data of specific treatment arms) (40).

## Results

In total, 23,267 records were initially retrieved from the electronic database search; of these, 12,361 duplicate records were removed. Among the remaining records, 10,829 were excluded based on a review of either the title or abstract and 77 records were retrieved for full-text review. Among these studies, 54 were excluded based on the following criteria: contained wrong dose of medication (*n* = 7), duplicated data (*n* = 9), contained patients with T2D (*n* = 17), review articles (*n* = 7), contained patients with liver cirrhosis and on dialysis (*n* = 4), contained adults with latent autoimmune diabetes (*n* = 5), editorial comment (*n* = 3), and failed to extract subject event (*n* = 2) (Figure 1).

Finally, 23 trials reporting outcomes for 5,151 patients (2,610 women and 2,541 men) were included in the analysis (Table 1). The average study duration was 30.8 ± 14.5 weeks. The trials were conducted in the following countries: the United States (1, 9, 11, 18, 23, 48, 51, 52) Denmark (12, 13, 42, 44), Canada (21, 41), Italy (46, 49), Austria (20), Belgium (14), Chile (47), France (45), Germany (50), India (10), and United Kingdom (1 each) (43). The number of patients per study ranged from 12 to 1,402, and the mean follow-up period was 17.01 (range, 11.5–38.0) years (Table 1).

**Table 1:** Important characteristics of the included studies and proportions of patients with using type 1 treatment.

**References**	**Country/year**	**Added Treatment/Dose**	**Number of patients (n I/C)**	**Age Mean ± SD ()median range, years**	**Male *n* (%)**	**Mean baseline BMI, kg/m^**2**^ mean (SD)**	**Mean baseline HbA1c, % mean (SD)**	**Mean baseline weight (kg)**	**Mean baseline Insulin dose, unit/kg/d**	**Mean duration of diabetes (year)**
(10)	India/2013	Exenatide/ 10 μg	6/6	28.8 ± 7.6		21.5 ± 1.5	9.7 ± 0.8	56.2 ± 3.4	55.7 ± 2.9	29.6 ± 8.8
(12)	Denmark/2015	Liraglutide/ 1.2 mg	18/18	39.5 ± 2.7	21/(58)	24.2 ± 0.6	8.8 ± 0.2	75.8 ± 2.9	62 ± 3.1	18.3 ± 2.0
(11)	USA/2016	Liraglutide/ 1.2 mg	16/17	42 ± 3	17/(52)	33 ± 2	7.8 ± 0.2	96.0 ± 4.0	71.2 ± 5.5	21 ± 3.0
(41)	Canada/2003	Metformin/ 500–2,000 mg	14/13	15.7 ± 1.9	12/(44.4)	29.5 ± 2.7	9.4 ± 1.0	62.9 ± 13.7		9.7 ± 4.4
(42)	Denmark/2008	Metformin/ 500–2,000 mg	12/12	43.5 ± 13.1	14/(58)	24.2 ± 0.6	8.9 ± 0.1	87.6 ± 2.7	62.7 ± 3.1	17.8 ± 10.3
(43)	UK/2006	Metformin/ 500–2,000 mg	15/15	48 ± 12	16/(53.3)	31.3 ± 2.6	8.6 ± 1.4	92 ± 12	60 ± 14	19 ± 10
(44)	Denmark/2008	Metformin/ 500–2,000 mg	47/45	46.1 ± 11.6	64/(69.5)	26.2 ± 3.4	9.5 ± 0.9	80.5 ± 12.5	59.8 ± 0.74	5 ± 0.51
(45)	France/2002	Metformin/ 850–1,500 mg	31/31	39.9 ± 12.9	37/(59.6)	26.4 ± 4.6	7.58 ± 0.84	78.4 ± 18.1	0.7 ± 0.2^*^	16.9 ± 8.9
(46)	Italy/2013	Metformin/ 850–1,500 mg	21/21	46 ± 8	18/(42.8)	28.7 ± 2.1	7.2 ± 0.9	83 ± 12	0.61 ± 0.22^*^	9.2 ± 0.7
(47)	Chile/2013	Metformin/ 850–1,500 mg	13/11	17.7 ± 1.6		23.7 ± 3.0	10.3 ± 2.3		1.2 ± 0.4	9.3 ± 5.1
(48)	USA/2015	Metformin/ 500–2,000 mg	71/69	15.4 ± 1.7	42/(34.2)	24.2 ± 0.6	8.8 ± 0.4	77 ± 6	1.1 ± 0.1^*^	7.0 ± 3.3
(49)	Italy/2015	Metformin/ 1,000 mg	15/13	15.0 ± 2.5	13/(46.4)	28.2 ± 6.6	9.3 ± 1.5	75.5 ± 25	84.0 ± 42.9	5.7 ± 4.4
(9)	USA/2014	Exenatide/ 10 μg	6/6	37.3 ± 10.7	11/(61)	26.1 ± 3.5	7.0 ± 0.8	77.7 ± 11.0	0.6 ± 0.1^*^	20.5 ± 11.8
(20)	Austria/2015	Empagliflozin/ 10 mg	19/19	39.6 ± 11.6	28/(73.6)	27.4 ± 3.5	8.3 ± 0.8	87.1 ± 13.3	0.7 ± 0.2	16.2 ± 8.4
(1)	USA/2017	Sotagliflozin/ 400 mg	699/703	43.3 ± 14.2	697/(49.7)	28.3 ± 5.1	8.3 ± 0.9	82.4 ± 17.1	56.9 ± 27.6	20.5 ± 12.4
(50)	Germany/2018	Sotagliflozin/ 400 mg	263/258	41.7 ± 13.23	250/(49.4)	29.6 ± 5.3	7.6 ± 0.7	86.5 ± 18.0	64.1 ± 37.6	24.4 ± 12.8
(51)	USA/2018	Sotagliflozin/ 400 mg	262/268	46.4 ± 13.1	257/(48.4)	24.2 ± 0.6	8.8 ± 0.2	75.8 ± 2.9	62 ± 3.1	18.3 ± 2.0
(52)	USA/2014	Sotagliflozin/ 400 mg	16/17	42.5 (21,55)	16/(48.4)	26.2 ± 3.0	7.9 ± 0.6	74.2(55.6, 107.9)	0.6^*^	16.8(3.4, 42.9)
(18)	USA/2018	Dapagliflozin/ 10 mg	296/260	42.7 ± 14.1	262/(50.5)	28.2 ± 5.2	8.5 ± 0.6	82.1 ± 17.4	59.4 ± 28.2	19.9 ± 11.1
(23)	USA/2014	canagliflozin/ 300 mg	117/117	42.8 ± 11.0	128/(54.4)	28.1 ± 3.9	8.0 ± 0.5	82.9 ± 15.0		21.9 ± 10.6
(14)	Belgium/2016	Liraglutide/ 1.2 mg	346/347	43.9 ± 13.1	346/(50.6)	29.3 ± 5.1	8.2 ± 0.8	85.4 ± 17.2	59.6 ± 49.8	21.6 ± 12.2
(21)	Canada/2018	Empagliflozin/ 10 mg	243/239	45.7 ± 12.5	227/(47.0)	29.5 ± 5.5	8.1 ± 0.6	86.2 ± 18.2	0.7 ± 0.2^*^	22.8 ± 12.6
(13)	Denmark/2016	Liraglutide/ 1.8 mg	50/50	47 ± 13	65/(65)	30.3 ± 3.5	8.7 ± 0.7	93.4 ± 14.2	32 ± 16	20 ± 12

*I, intervention group; C, Control group*;

* * = (units _kg_1 _day_1); UK, United Kingdom; USA, United States of America*.

### Risk of Bias in the Included Studies

Although all included studies were described as randomized, a few studies provided specific details of either the method of randomization or concealment of allocation. For all the included studies, blinding had been done adequately.

### Effect of Interventions

**Figure 3:**
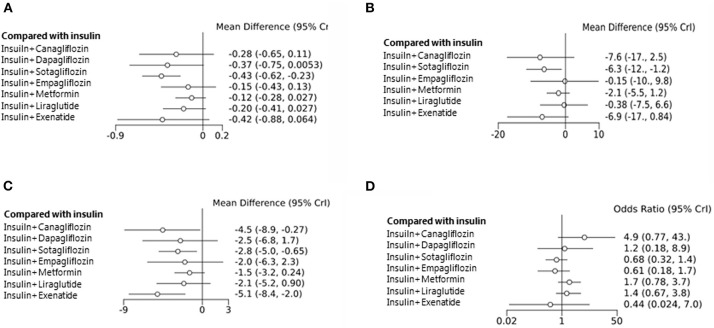
Mean change in HbA1c level from the baseline **(A)**. Mean change in daily insulin dose from the baseline **(B)**. Mean change in body weight from the baseline **(C)**. Hypoglycemic events **(D)** associated with different types of treatment compared with the placebos used as the reference.

Data obtained from all the 23 studies (*n* = 5,151) were subjected to the network analysis. The primary endpoint was a change in HbA1c level. Compared with the insulin alone treatment as the reference, sotagliflozin treatment significantly reduced the HbA1c level (MD: −0.43, 95% CrI: −0.62 to −0.23) (Figure 3A). However, canagliflozin (−0.28, 95% CrI:−0.65 to 0.11), dapagliflozin (−0.37, 95% CrI: −0.75 to 0.01), empagliflozin (−0.15, 95% CrI: −0.43 to 0.13), metformin (−0.12, 95% CrI: −0.28 to 0.03), liraglutide (−0.20, 95% CrI: −0.41 to 0.03), and exenatide (−0.42, 95% CrI: −0.88 to 0.06) showed no significant changes in HbA1c compared with insulin alone. Among the studies with sotagliflozin, a study by Sands et al. had a noticeably short study treatment duration (29 days) (52). The sensitivity analysis was performed after excluding this study and showed that sotagliflozin therapy reduced HbA1c level significantly (Supplementary Figure 4 and Supplementary Tables 2, 3).

We further analyzed the total insulin daily dose (TIDD), weight change, and adverse effects as the secondary endpoints. Among the eight studied agents, sotagliflozin decreased the TIDD compared with insulin alone, whereas the other drugs showed no change in the TIDD (MD: −6.3 IU, 95% CrI: −12 to −1.20) (Figure 3B). A decrease in body weight from the baseline was observed after treatment with canagliflozin (−4.5 kg, 95% CrI: −8.90 to −0.27), sotagliflozin (−2.8, 95% CrI: −5.0 to −0.65), and exenatide (−5.1, 95% CrI: −8.4 to −2.0) (Figure 3C). However, the frequency of hypoglycemia was not significantly different among the intervention groups (Figure 3D).

Diabetic ketoacidosis (DKA) is one of the most serious adverse effects observed in patients with type 1 diabetes. In the present study, DKA was more frequently observed with canagliflozin (OR = 18.0, 95% CrI: 1.5 to 6.7e+0.2) and sotagliflozin (OR = 6.9, 95% CrI: 2.0 to 29.0) treatments.

Other adverse events, such as myocardial infarction, heart failure, stroke, hospitalization, peripheral artery disease, diarrhea, pancreatitis, renal event, and urinary tract infection or genital infection, did not differ among the medication groups. These side effects were similar to those of only sotagliflozin subgroup, which showed the best effect. Compared to placebo, the event of acidosis, defined as lactic acidosis, metabolic acidosis, renal tubular acidosis, and uremic acidosis, in the sotagliflozin group was approximately 2.82 times higher (Odds ratio: 2.82, 95% CI: 1.87 to 4.26). Using meta-analysis, diabetic ketoacidosis (DKA) was also observed in patients, more frequently with sotagliflozin (OR = 5.91, 95% CI: 2.45 to 14.2) treatment group (Supplementary Figure 5).

### Rank Probabilities

**Figure 4:**
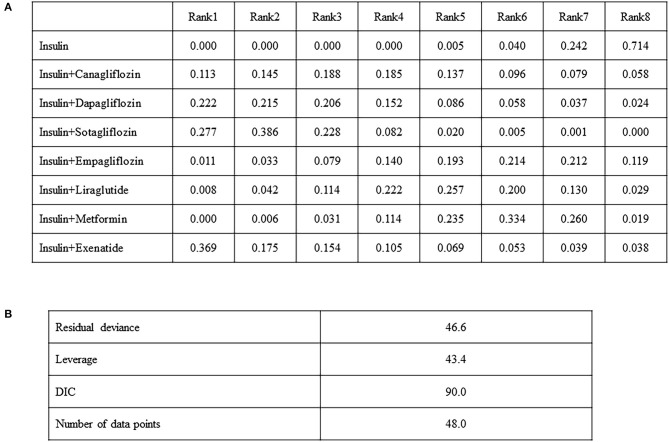
Comparison of the included diabetes treatments for HbA1c, odds ratio (95% CI). Each cell indicates the effect of the column-defining intervention relative to the row-defining intervention **(A)**, model fit statistics **(B)**.

In terms of changes in the HbA1c level as the primary outcome, network meta-analysis can statistically rank the outcomes by measuring their probability. Figure 4A shows several probabilities in Rank 1, indicating that the interventions will be ranked first in the network flow. The highest probability of insulin exenatide was 0.369 but was not statistically significant. However, the next probability, insulin sotagliflozin, was statistically significant in the network meta-analysis, and the probability of being first in the rankogram was 0.277, which was the highest reduction in the HbA1c level among the treatments (Figure 4A). Model fit was assessed by comparing deviance information criterion and residual deviance. Deviance information criterion (DIC) measures the deviance, estimated by the posterior mean of minus twice the log-likelihood plus the effective number of parameters in the model. The DIC measures the model fit that penalizes model complexity—lower DIC values suggest a more parsimonious model. The DIC and residual deviance for HbA1c were 90.0 and 46.6, respectively (Figure 4B). The model that analyzed HbA1c showed a small DIC number of <150, therefore, the change was minimized, and the model could be selected as an appropriate model.

The rank probabilities of mean change in body weight from the baseline for add-on drugs were in the following order: exenatide (0.482), canagliflozin (0.290), and sotagliflozin (0.169) (Supplementary Figure 6A). Model fit statistic of DIC of any weight reduction was 63.2 and the residual deviance was 33.8 (Supplementary Figure 7A).

## Discussion

In the present study, we evaluated the effect and safety of adding oral hypoglycemic agents (metformin, SGLT2 inhibitor, or SGLT1/2 co-inhibitor) or injectable GLP-1 RAs to insulin therapy in patients with T1D. Among these agents, sotagliflozin add-on therapy was found to be the most effective in reducing HbA1c levels. Treatment with canagliflozin, sotagliflozin, and exenatide decreased the body weight (a secondary outcome) by 4.5, 2.8, and 5.1 kg, respectively. The TIDD was significantly decreased in the sotagliflozin treatment group (6.3 IU/day) compared with that in the insulin monotherapy group. Hypoglycemic episodes and other adverse events did not differ between the groups. These data suggest that sotagliflozin and short-acting GLP-1RA and SGLT inhibitor add-on therapies could have beneficial effects in lowering the HbA1c level, insulin dose, and body weight in patients with T1D undergoing insulin treatment.

In previous clinical trials, early intensive glucose control in patients with T1D was reported to reduce all-cause mortality and prevent or delay late microvascular and macrovascular complications of diabetes (53). Therefore, insulin therapy is essential for T1D, but weight gain is a major concern. According to an analysis of physician electronic health records in the United States, 47.8% of people with T1D were found to be obese (54). Obesity is associated with insulin resistance and increased cardiovascular complications. Patients with T1D with more than two complications have significantly higher BMI than those with less than one complication (55).

Another important issue in the management of T1D is glycemic variability. A recent study demonstrated that variability in the HbA1c level was significantly and additively associated with mortality in participants (>13 years old) with T1D (56). Potential underlying mechanisms are unclear, but the variability in HbA1c could result in a poor response to insulin therapy or hypoglycemia. In a study on 1,706 adolescents with T1D, HbA1c variability significantly increased the risk of retinopathy, albuminuria, and cardiac autonomic neuropathy (57). Oxidative stress and systemic inflammation induced by inflammatory cytokines have been hypothesized as a potential mechanism underlying the association between glycemic variability and increased risk of diabetic complications (58, 59).

Taken together, intensive insulin treatment is essential for preventing diabetic complications caused by hyperglycemia, but it is associated with adverse effects, such as weight gain, hypoglycemia, and hyperglycemia, which cause low compliance leading to glycemic variability. In addition, the risk of hyperglycemia or hypoglycemia decreases the quality of life of patients with T1D (60).

Therefore, considerable effort has been made for better glycemic control without hypoglycemia using new antidiabetic medications. SGLT2 inhibitors target the proximal tubular SGLT2 transport protein, which is responsible for ~90% of renal glucose reabsorption (16). Glucosuria caused by SGLT2 inhibition can result in a caloric loss of 250–300 kcal/day and, consequently, a weight loss of 2–3 kg. Given their insulin-independent mechanism, SGLT2 inhibitors have been used in several studies on T1D. In the Empagliflozin as Adjunctive to Insulin Therapy-2 and−3 studies, empagliflozin add-on to insulin improved glycemic control and weight change without increasing hypoglycemia in patients with T1D (21). However, adjudicated DKA occurred more frequently with 10 mg (4.3%) and 25 mg empagliflozin (3.3%). In the DEPICT-1 study, dapagliflozin treatment significantly reduced HbA1c by 0.42–0.45%, body weight by 2.96–3.72%, and TIDD by 8.8–13.2% after 24 weeks (19). DKA rates were higher after dapagliflozin treatment than after placebo treatment. Canagliflozin treatment also showed a similar efficacy in HbA1c reduction and body weight control, but the incidence of DKA requiring hospitalization was significantly increased with canagliflozin treatment compared with placebo treatment (22).

Sotagliflozin is a novel dual inhibitor of SGLT1 and SGLT2 that can reduce glucose absorption in the proximal intestine. SGLT1 inhibition was shown to increase the delivery of glucose to the distal small intestine and augment GLP-1 release (16, 61). In a phase III RCT of sotagliflozin administered in combination with insulin to 1,402 adults with T1D, 24 weeks of treatment with sotagliflozin decreased the HbA1c level by 0.46%, body weight by 2.98 kg, and insulin dose by 2.8 U/day (1). However, sotagliflozin treatment was associated with a higher rate of ketoacidosis (3.0%) than placebo (0.6%).

The present study data revealed that sotagliflozin add-on to insulin improved glycemic control and decreased weight. These positive effects may be related to an improvement in glucose variability. Although the clinical role of SGLT1 inhibition at therapeutic doses is unlikely, the beneficial effects of sotagliflozin on glycemic improvement and weight loss in the present study suggest that a more marked inhibition of SGLT1 should be explored.

Canagliflozin, which also inhibits SGLT1, was also effective in body weight reduction in patients with T1D. A study showed that canagliflozin inhibited intestinal glucose absorption at a concentration 10-times the IC50 of SGLT1 in the intestinal lumen (62). A recent randomized trial in patients with type 2 diabetes showed that pre-meal administration of canagliflozin increased the plasma GLP-1 levels (63). These findings suggest that canagliflozin has a positive role in weight reduction in patients with T1D.

A brief report on cardiovascular effects of exenatide add-on therapy in 69 metformin-treated patients with T2D showed a significant reduction in total body fat mass, trunk fat mass, and waist circumference compared with the insulin glargine therapy. According this study, treatment with exenatide for 1 year reduced body weight (6%), waist circumference (5%), and total body (11%) and truncal fat mass (13%) (64).

A recent prospective, randomized study investigated exenatide or glargine add-on therapy in 37 overweight or obese patients with T2D, who were inadequately treated with metformin. After 16 weeks, the exenatide treatment group had lower body weight (−4.5 kg), BMI (−1.6 kg/m^2^), body fat mass, and percent fat mass (except for gynoid fat) than the insulin glargine group. Weight loss by exenatide was mainly owing to reduced body fat content rather than lean tissue (65).

A study on metformin-treated patients with T2D showed that 1 year treatment with exenatide reduced the total body fat mass by 6% and the waist circumference by 5% compared with the insulin glargine-treated patients (37). In another recent prospective, randomized study in overweight or obese patients with T1D, 16 weeks of treatment with exenatide significantly decreased the body weight by 4.5 kg and BMI by 1.6 kg/m^2^ compared with insulin glargine treatment (38). Moreover, exenatide resulted in weight loss mainly by reducing body fat but not lean tissue mass. The findings of our study suggest that sotagliflozin has potential benefits of HbA1c reduction and weight loss, whereas canagliflozin and exenatide have a potential benefit of weight loss in patients with T1D.

Our study had several strengths. A traditional meta-analysis can compare only two groups based on one intervention, which is a limitation. However, our network meta-analysis is a complement method to the groups, interventions, or conflict interests that are difficult to be directly compared with each other. In this study, we performed an indirect analysis that can explain the accuracy of model by 20,000 repetitive learning in computer and rank among interventions by comparing several groups at the same time. This is pivotal to guideline development for T1D since, in the absence of head-to-head evidence, guideline development groups will rely more strongly on expert opinion. Hence, they may make comparisons that do not adequately account for potential biases in study designs, intervention characteristics, and study populations. Indirect comparisons connect treatments via a common control or comparator (e.g., a placebo like insulin or a standard of care) thus having a comparative effect between treatments that have not been compared head-to-head in randomized controlled trials. Another benefit of this analysis is that it comprises a simultaneous analysis of all potential treatment options and makes full use of the available evidence within a single analysis. Doing so, provides a more concise assessment of the clinical landscape and enables better decision-making. This analysis can be helpful in selecting add-on drugs for a specific condition. Secondly, this is the first network meta-analysis involving the SGLT1/2 inhibitor, sotagliflozin. Finally, we exclusively included well-designed RCTs. Therefore, less accurate studies were excluded, and the results were less biased to increase reliability.

Our study also had some limitations. Some of the trials included had a relatively small number of participants, and they were conducted for a short duration. Thus, the assessment of long-term outcomes such as cardiovascular events and renal complications was not performed. Another limitation is that the sotagliflozin study included HbA1c reduction results with an insufficient drug duration, which may weaken the findings. However, the sensitivity analysis proved that sotagliflozin therapy reduced HbA1c level significantly in patients with T1D.

In conclusion, sotagliflozin treatment decreased not only the HbA1c levels and insulin dose but also the body weight without causing hypoglycemia in patients with T1D. Treatment with canagliflozin and exenatide was effective in body weight reduction in patients with T1D. However, when we performed a meta-analysis using only four studies, including sotagliflozin, the sotagliflozin group had an increased risk of acidosis and diabetic ketoacidosis compared to the placebo. Therefore, adverse effects associated with SGLT inhibitors should be considered in these patients.

In March 2019, the US FDA rejected the use of sotagliflozin as an adjunct to insulin for the treatment of T1D. The decision followed a split vote in January 2019 by the FDA's Endocrinologic and Metabolic Drugs Advisory Committee, during which panel members expressed concerns over an increased risk of ketoacidosis with the drugs used for T1D. However, after 1 month, the European Commission approved sotagliflozin for prescription in the European Union for certain overweight patients with T1D. According to the results of our network meta-analysis, we suggest that sotagliflozin has a high probability of being ranked first as an adjunctive therapy to insulin in patients with T1D. However, to avoid ketoacidosis and other adverse events, risk mitigation strategies, such as continuation of insulin and discontinuation of SGLT inhibitors on sick days, should be strictly implemented when these drugs are introduced for patients with T1D (66).

## Data Availability Statement

All datasets presented in this study are included in the article/Supplementary Material.

## Author Contributions

YK, SH, and SL: conceptualization, methodology and data acquisition, data analysis and interpretation, statistical analysis, writing—original draft preparation, writing—review and editing, and funding acquisition. All authors contributed to the article and approved the submitted version.

## Conflict of Interest

The authors declare that the research was conducted in the absence of any commercial or financial relationships that could be construed as a potential conflict of interest.
