# Supplementary figures and images for: Effects of Sodium-Glucose Cotransporter Inhibitor/Glucagon-Like Peptide-1 Receptor Agonist Add-On to Insulin Therapy on Glucose Homeostasis and Body Weight in Patients With Type 1 Diabetes: A Network Meta-Analysis
Source: Front Endocrinol (Lausanne). 2020 Aug 19;11:553. doi: 10.3389/fendo.2020.00553 (PMC7466678; doi:10.3389/fendo.2020.00553)

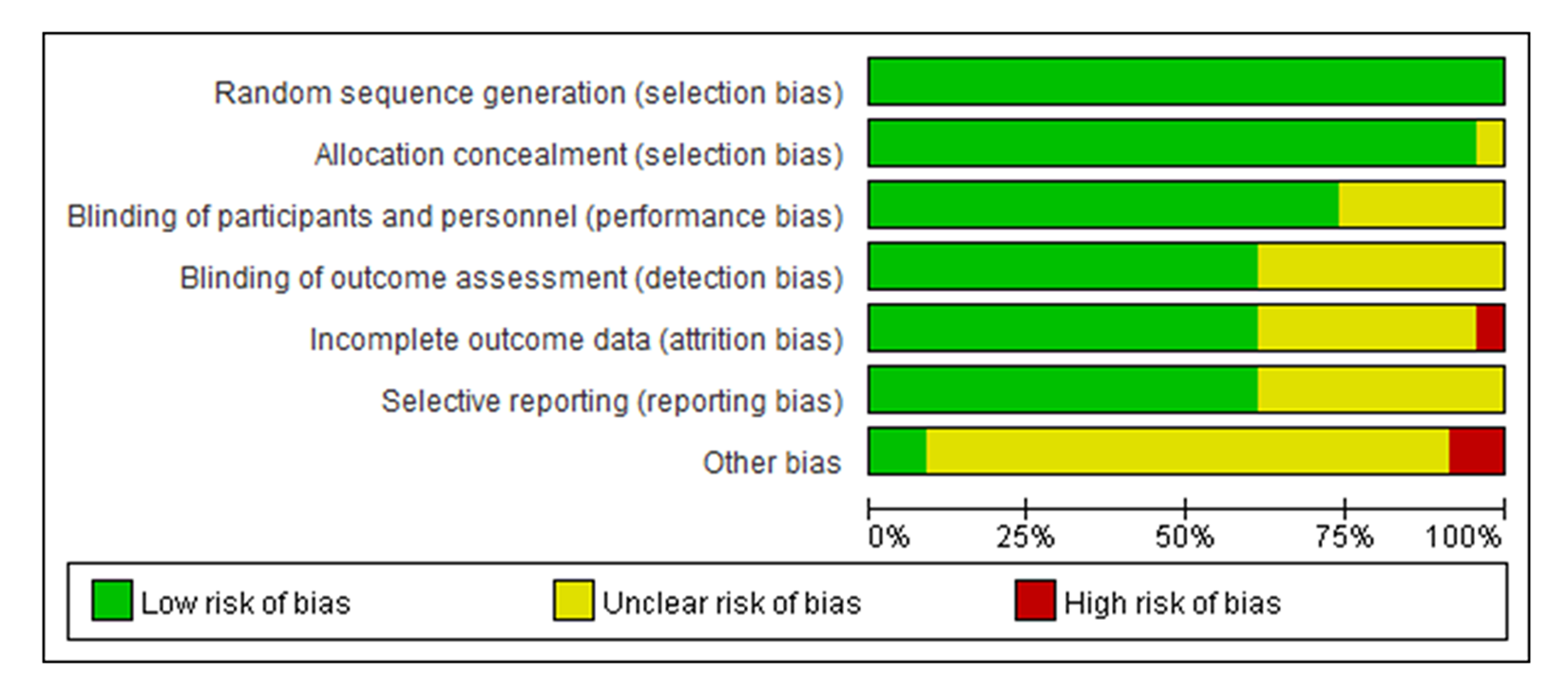

Supplement: Supplementary file 1 [file Image_1.TIF]

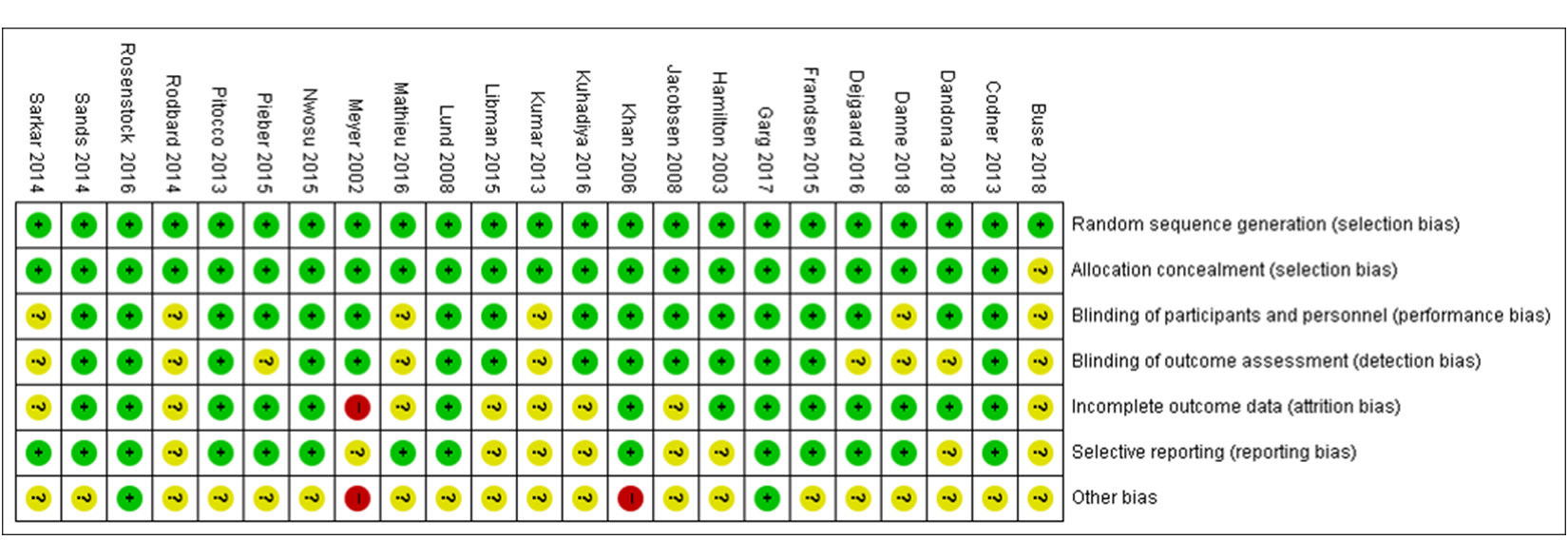

Supplement: Supplementary file 2 [file Image_2.TIF]

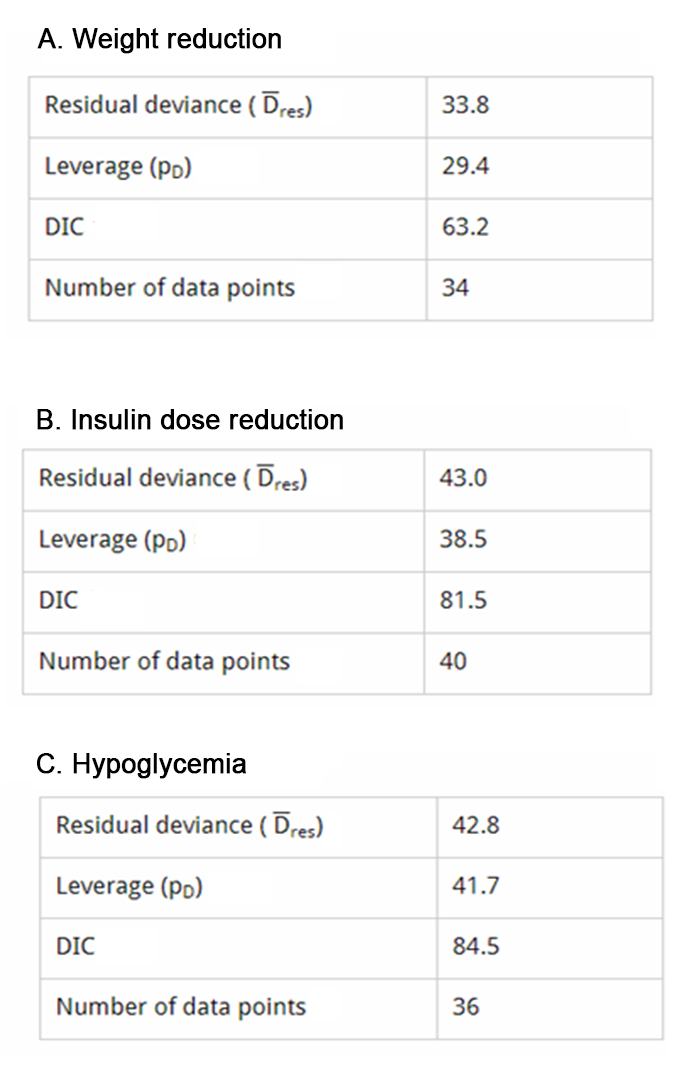

Supplement: Supplementary file 3 [file Image_3.TIF]

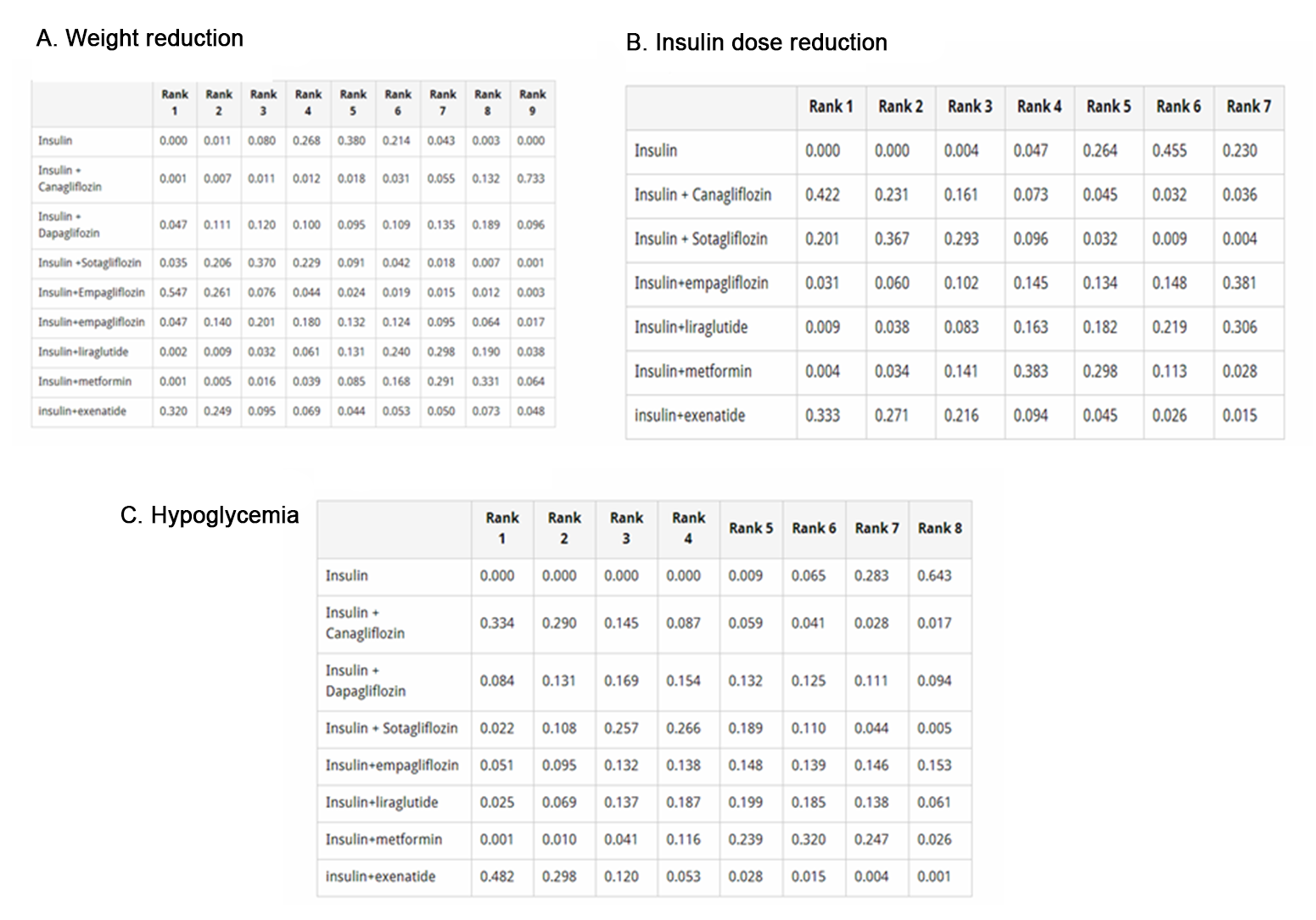

Supplement: Supplementary file 4 [file Image_4.TIF]

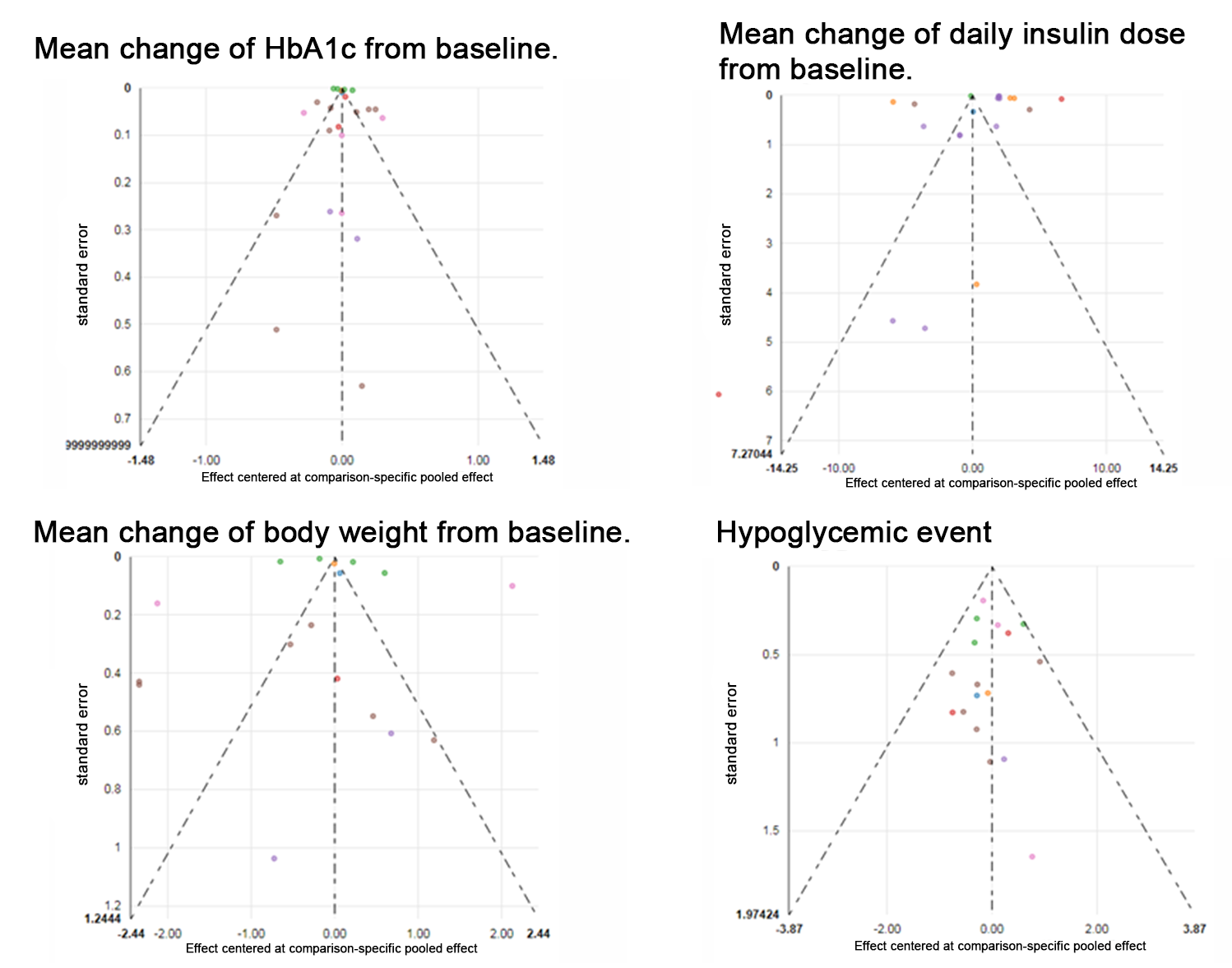

Supplement: Supplementary file 5 [file Image_5.TIF]

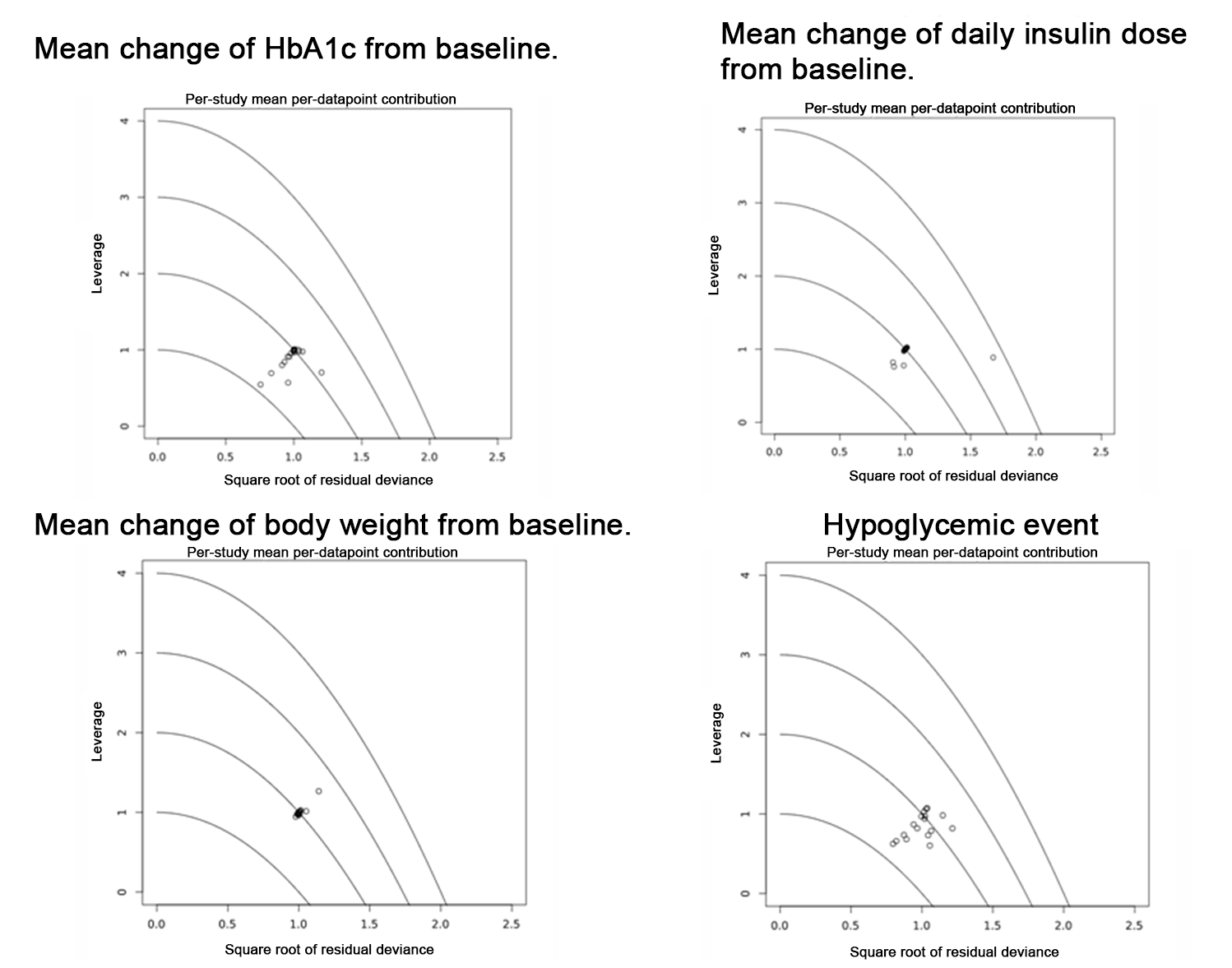

Supplement: Supplementary file 6 [file Image_6.TIF]

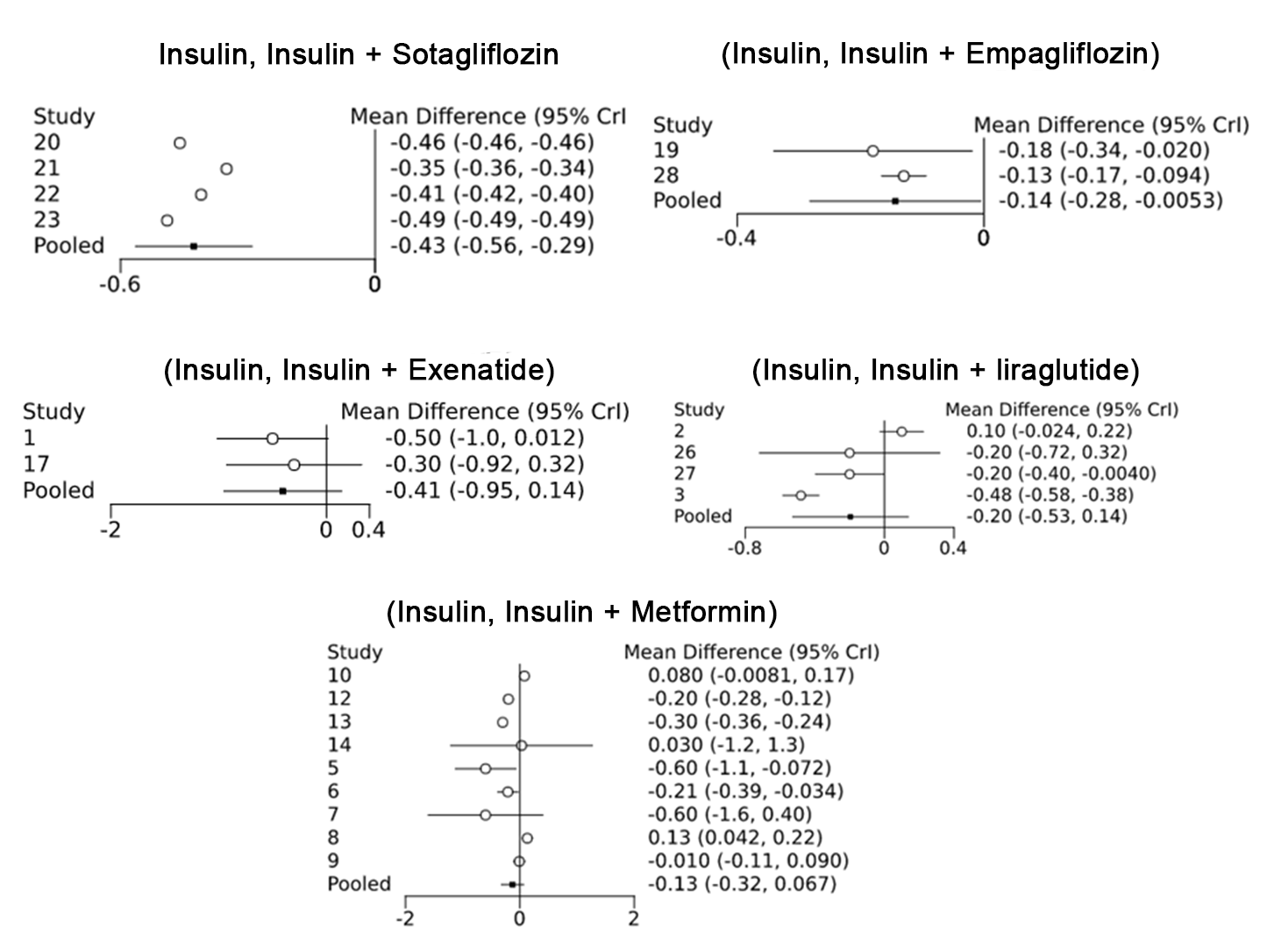

Supplement: Supplementary file 7 [file Image_7.TIF]

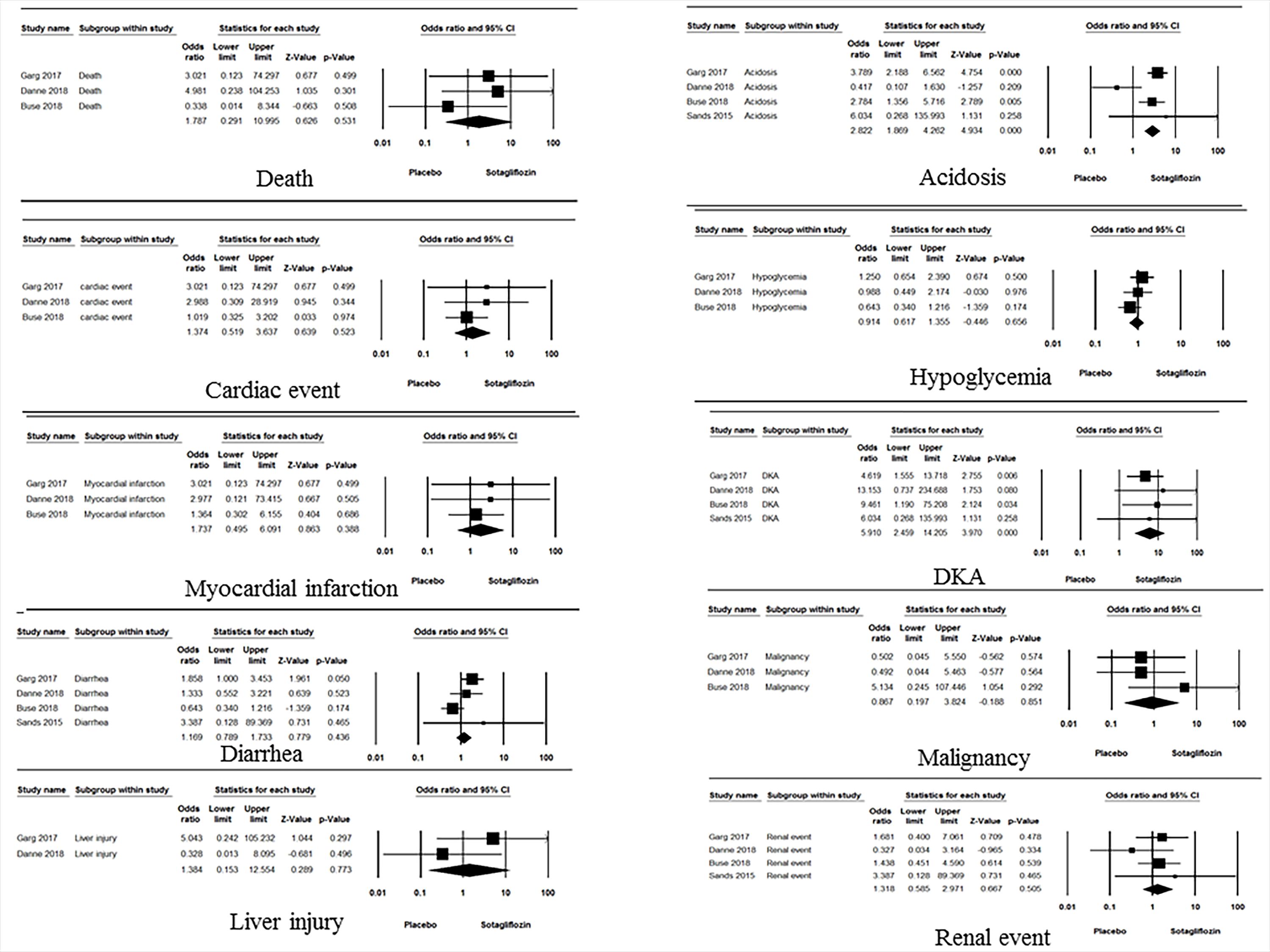

Supplement: Supplementary file 8 [file Image_8.TIF]

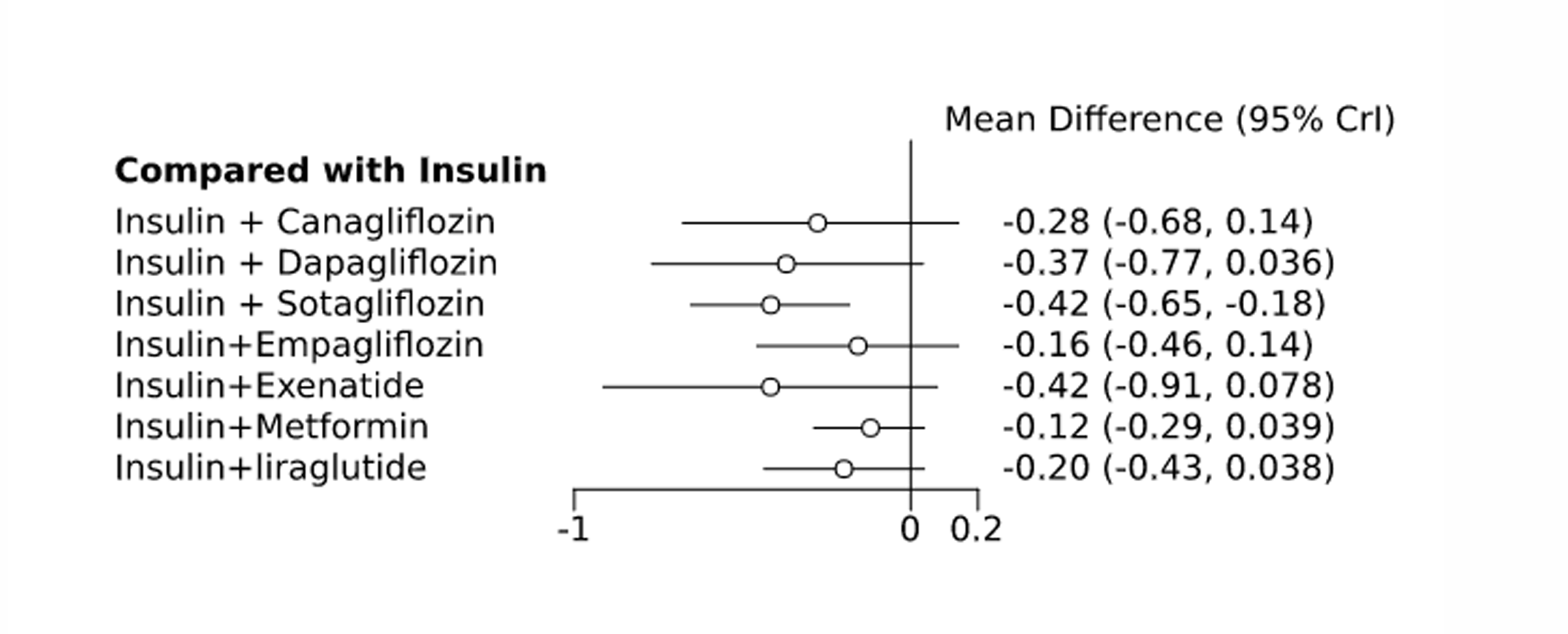

Supplement: Supplementary file 9 [file Image_9.TIF]
