# Supplementary material for: Effects of Sodium-Glucose Cotransporter Inhibitor/Glucagon-Like Peptide-1 Receptor Agonist Add-On to Insulin Therapy on Glucose Homeostasis and Body Weight in Patients With Type 1 Diabetes: A Network Meta-Analysis
Source: Front Endocrinol (Lausanne). 2020 Aug 19;11:553. doi: 10.3389/fendo.2020.00553 (PMC7466678; doi:10.3389/fendo.2020.00553)
Supplement: Supplementary file 10 [file Data_Sheet_1.docx]

Supplementary Material

Supplementary Data

Supplementary information 1. Identified studies through an electronic databases search using various search terms.

**Supplementary Table 2** Comparison of the included interventions in sensitivity analysis: mean difference (95% CrI). Each cell gives the effect of the column-defining intervention relative to the row-defining intervention

**Supplementary Table 3.** Rank probabilities table with sensitivity analysis

Supplementary Figures

Supplementary Figure 1. Risk of bias graph. Assessment of each risk of bias item is presented as a cumulative percentage of all included studies.

Supplementary Figure 2. Risk of bias graph and summary assessment of each risk of bias item for each included study. Plus green circle, good; yellow circle, moderate; minus red circle, bad.

Supplementary Figure 3. Model fit statistics for each study. Mean change in body weight from that at baseline (A), mean change of daily insulin dose from that at baseline (B), and hypoglycemic events (C).

Supplementary Figure 4. Comparison of the included diabetes treatments for each outcome; odds ratio (95% CI). Each cell indicates the effect of the column-defining intervention relative to the row-defining intervention.

Supplementary Figure 5. Funnel plot of study effects according to each outcome.

Supplementary Figure 6. Leverage versus residual deviance per-study mean per-data point contribution of each result.

Supplementary Figure 7*.* Results of traditional pair-wise meta-analysis of HbA1c levels.

Supplementary Figure 8*.* Results of a traditional meta-analysis of the adverse effects of sotagliflozin.

**Supplementary Figure 9**. Mean change in HbA1c level from the baseline associated with different types of treatment compared with the placebos used as the reference in sensitivity analysis.
