# Supplementary material for: Effects of Sodium-Glucose Cotransporter Inhibitor/Glucagon-Like Peptide-1 Receptor Agonist Add-On to Insulin Therapy on Glucose Homeostasis and Body Weight in Patients With Type 1 Diabetes: A Network Meta-Analysis
Source: Front Endocrinol (Lausanne). 2020 Aug 19;11:553. doi: 10.3389/fendo.2020.00553 (PMC7466678; doi:10.3389/fendo.2020.00553)
Supplement: Supplementary file 11 [file Table_1.docx]

**Supplemt 1. Detailed Search term for selection of study**

((((((((((((((((((((((((((((((((((( agonists, amylin receptor OR receptor agonists, amylin OR amylin receptor agonist OR agonist, amylin receptor OR receptor agonist, amylin OR amylin agonists OR agonists, amylin OR amylin mimetics OR mimetics, amylin))) OR ((glucagon like peptide 1 OR GLP-1 OR GLP 1 OR glucagon-like peptide-1))) OR (victoza OR saxena OR NN 2211 OR 2211, NN OR NN2211 OR NN-2211)) OR ((bydureon OR itca 650 OR AC 2993 LAR OR Exendin-4 OR ex4 peptide OR peptide, ex4 OR exendin 4 OR byetta OR AC 2993))) OR (( dipeptidyl peptidase iv inhibitors OR inhibitors, dipeptidyl-peptidase iv OR gliptins OR dipeptidyl-peptidase 4 inhibitors OR dipeptidyl peptidase 4 inhibitors))) OR (((2S) (((3-Hydroxyadamantan-1-yl) amino) acetyl) pyrrolidine-2-carbonitrile OR NVP-LAF237 OR NVP LAF237 OR gilvus))) OR ((phosphate, sitagliptin OR sitagliptin phosphate monohydrate OR monohydrate, sitagliptin phosphate OR phosphate monohydrate, sitagliptin OR sitagliptin monophosphate monohydrate OR monohydrate, sitagliptin monophosphate OR monophosphate monohydrate, sitagliptin OR MK 0431 OR 0431, MK OR MK0431 OR MK-0431 OR sitagliptin phosphate anhydrous OR anhydrous, sitagliptin phosphate OR phosphate anhydrous, sitagliptin OR sitagliptin OR 4-Oxo-4- (3- (trifluoromethyl) -5,6-dihydro (1,2,4) triazolo (4,3-a) pyrazin-7 (8H) -yl) -1- (2,4,5-trifluorophenyl) butan-2-amine OR januvia))) OR (( sodium glucose transporter 2 inhibitors OR sglt2 inhibitors OR sglt-2 inhibitors OR sglt 2 inhibitors OR gliflozins))) OR (invokana OR canagliflozin hemihydrate OR canagliflozin, anhydrous)) OR (( sodium glucose transporter 1 OR slc5a1 protein OR sodium-glucose cotransporter 1 OR sodium glucose cotransporter 1 OR sglt1 protein))) OR ((sotagliflozin OR LX4211 OR LX-4211))) OR ((sodium glucose transporter 2 OR slc5a2 protein OR sglt2 protein))) OR (( sodium glucose transport proteins OR glucose sodium cotransport system OR sglt proteins OR sodium glucose transport system OR sodium glucose transporters OR glucose transporters, sodium OR sodium-glucose transport system OR glucose sodium cotransport system OR sodium glucose cotransporters OR glucose cotransporters, sodium OR sodium-sugar transporter OR sodium sugar transporter OR transporter, sodium-sugar))) OR ( sodium glucose transport proteins OR glucose sodium cotransport system OR sglt proteins OR sodium glucose transport system OR sodium glucose transporters OR glucose transporters, sodium OR sodium-glucose transport system OR glucose sodium cotransport system OR sodium glucose cotransporters OR glucose cotransporters, sodium OR sodium-sugar transporter OR sodium sugar transporter OR transporter, sodium-sugar)) OR ((dimethylbiguanidine OR dimethylguanylguanidine OR glucophage OR metformin hydrochloride OR hydrochloride, metformin OR metformin hcl OR hcl, metformin)))))) OR ((eperzan OR tanzeum OR albiglutide OR albiglutide))) OR ((LY 2189265 OR LY-2189265 OR LY2189265 OR trulicity OR dulaglutide))) OR ((semaglutide OR taspoglutide))) OR ((lixisenatide OR DES-38-proline-exendine-4 (helodermasuspectum) (1-39) peptidylpenta l lysyl l lysinamide OR adlyxin OR AQVE-10010 OR zp10a peptide OR ZP 10 OR ZP-10 OR lyxumia OR AVE 010 OR AVE-010 OR AVE 0010 OR AVE0010 OR AVE-0010))) OR ((alogliptin OR 2- ((6- ((3R) -3-aminopiperidin-1-yl) -3-methyl-2,4-dioxo-3,4-dihydropyrimidin-1 (2H) -yl) methyl) benzonitrile OR nesina OR SYR 322 OR SYR322 OR SYR-322))) OR canagliptin) OR gemigliptin) OR ((linagliptin OR R) -8- (3-amino-piperidin-1-yl) -7-but-2-ynyl-3-methyl-1- (4-methyl-quinazolin-2-ylmethyl) -3,7-dihydro-purine-2,6-dione OR 1H-purine-2,6-dione, 8- ((3r) -3-amino-1-piperidinyl) -7- (2-butynyl) -3,7-dihydro-3-methyl-1- ((4-methyl-2-quinazolinyl) methyl) BI 1356 OR 1356, BI OR BI1356 OR BI-1356 OR tradjenta OR tradjenta)) OR ((((omarigliptin OR MK-3102)) OR (saxagliptin OR 3-hydroxyadamantylglycine-4,5-methanoprolinenitrile hydrate OR onglyza OR BMS 477118 OR BMS477118 OR BMS-477118)) OR teneligliptin)) OR ((ertugliflozin OR ertugliflozin OR PF 04971729 OR PF04971729 OR PF-04971729))) OR ((tofogliflozin OR tofogliflozin hydrate OR CSG452 OR tofogliflozin anhydrous OR apleway OR deberza OR tofogliflozin))) OR ((ipragliflozin OR (1S) -1,5-anhydro-1- (3- (1-benzothiophen-2-ylmethyl) -4-fluorophenyl) d glucitol OR suglat OR ASP1941 OR ASP-1941))) OR (ipragliflozin OR (1S) -1,5-anhydro-1- (3- (1-benzothiophen-2-ylmethyl) -4-fluorophenyl) d glucitol OR suglat OR ASP1941 OR ASP-1941)) OR remogliflozin)) AND ((Type 1 Diabetes Mellitus OR Diabetes Mellitus, Type I OR Type 1 Diabetes OR Diabetes, Type 1 OR Diabetes Mellitus, Ketosis-Prone OR Diabetes Mellitus, Ketosis Prone OR Ketosis-Prone Diabetes Mellitus OR Diabetes, Autoimmune OR Autoimmune Diabetes OR Diabetes Mellitus, Juvenile-Onset OR Diabetes Mellitus, Juvenile Onset OR Juvenile-Onset Diabetes Mellitus OR Juvenile-Onset Diabetes OR Diabetes, Juvenile-Onset OR Juvenile Onset Diabetes OR Diabetes Mellitus, Insulin-Dependent OR Diabetes Mellitus, Insulin Dependent OR Insulin-Dependent Diabetes Mellitus OR IDDM OR Diabetes Mellitus, Insulin-Dependent OR Insulin-Dependent Diabetes Mellitus 1 OR Insulin Dependent Diabetes Mellitus 1 OR Diabetes Mellitus, Brittle OR Brittle Diabetes Mellitus OR Diabetes Mellitus, Sudden-Onset OR Diabetes Mellitus, Sudden Onset OR Sudden-Onset Diabetes Mellitus))
