# Supplementary material for: Effects of Sodium-Glucose Cotransporter Inhibitor/Glucagon-Like Peptide-1 Receptor Agonist Add-On to Insulin Therapy on Glucose Homeostasis and Body Weight in Patients With Type 1 Diabetes: A Network Meta-Analysis
Source: Front Endocrinol (Lausanne). 2020 Aug 19;11:553. doi: 10.3389/fendo.2020.00553 (PMC7466678; doi:10.3389/fendo.2020.00553)
Supplement: Supplementary file 12 [file Table_2.docx]

|  | | | | | | | |
| --- | --- | --- | --- | --- | --- | --- | --- |
| Insulin | -0.277 ( -0.677, 0.141) | -0.370 ( -0.769, 0.036) | -0.416 ( -0.652, -0.184) | -0.156 ( -0.456, 0.141) | -0.417 ( -0.912, 0.078) | -0.119 ( -0.287, 0.039) | -0.198 ( -0.435, 0.038) |
|  | Insulin + Canagliflozin | -0.094 ( -0.660, 0.481) | -0.141 ( -0.615, 0.325) | 0.120 ( -0.392, 0.619) | -0.143 ( -0.775, 0.504) | 0.160 ( -0.300, 0.579) | 0.078 ( -0.391, 0.535) |
|  |  | Insulin + Dapagliflozin | -0.048 ( -0.512, 0.412) | 0.214 ( -0.290, 0.722) | -0.048 ( -0.670, 0.583) | 0.252 ( -0.194, 0.671) | 0.173 ( -0.303, 0.636) |
|  |  |  | Insulin + Sotagliflozin | 0.262 ( -0.121, 0.634) | -0.001 ( -0.541, 0.551) | 0.299 ( 0.012, 0.570) | 0.219 ( -0.121, 0.550) |
|  |  |  |  | Insulin+Empagliflozin | -0.263 ( -0.830, 0.337) | 0.039 ( -0.306, 0.364) | -0.043 ( -0.423, 0.336) |
|  |  |  |  |  | Insulin+Exenatide | 0.296 ( -0.223, 0.814) | 0.220 ( -0.327, 0.759) |
|  |  |  |  |  |  | Insulin+Metformin | -0.077 ( -0.371, 0.214) |
|  |  |  |  |  |  |  | Insulin+liraglutide |
