# Supplementary material for: Effects of Sodium-Glucose Cotransporter Inhibitor/Glucagon-Like Peptide-1 Receptor Agonist Add-On to Insulin Therapy on Glucose Homeostasis and Body Weight in Patients With Type 1 Diabetes: A Network Meta-Analysis
Source: Front Endocrinol (Lausanne). 2020 Aug 19;11:553. doi: 10.3389/fendo.2020.00553 (PMC7466678; doi:10.3389/fendo.2020.00553)
Supplement: Supplementary file 13 [file Table_3.docx]

|  | | | | | | | | |
| --- | --- | --- | --- | --- | --- | --- | --- | --- |
|  | **Rank 1** | **Rank 2** | **Rank 3** | **Rank 4** | **Rank 5** | **Rank 6** | **Rank 7** | **Rank 8** |
| Insulin | 0.000 | 0.000 | 0.000 | 0.002 | 0.008 | 0.055 | 0.272 | 0.664 |
| Insulin + Canagliflozin | 0.105 | 0.150 | 0.183 | 0.188 | 0.135 | 0.104 | 0.066 | 0.068 |
| Insulin + Dapagliflozin | 0.230 | 0.230 | 0.193 | 0.143 | 0.087 | 0.054 | 0.034 | 0.029 |
| Insulin + Sotagliflozin | 0.259 | 0.351 | 0.234 | 0.106 | 0.034 | 0.013 | 0.003 | 0.001 |
| Insulin+Empagliflozin | 0.014 | 0.042 | 0.089 | 0.152 | 0.209 | 0.209 | 0.169 | 0.117 |
| Insulin+Exenatide | 0.379 | 0.175 | 0.145 | 0.102 | 0.066 | 0.052 | 0.039 | 0.042 |
| Insulin+Metformin | 0.001 | 0.006 | 0.031 | 0.092 | 0.197 | 0.322 | 0.303 | 0.049 |
| Insulin+liraglutide | 0.012 | 0.046 | 0.126 | 0.214 | 0.264 | 0.191 | 0.114 | 0.032 |
